# A Revisit to High Thermoelectric Performance of Single-layer MoS_**2**_

**DOI:** 10.1038/srep18342

**Published:** 2015-12-17

**Authors:** Zelin Jin, Quanwen Liao, Haisheng Fang, Zhichun Liu, Wei Liu, Zhidong Ding, Tengfei Luo, Nuo Yang

**Affiliations:** 1 State Key Laboratory of Coal Combustion, Huazhong University of Science and Technology (HUST), Wuhan 430074, People’s Republic of China; 2 Nano Interface Center for Energy (NICE), School of Energy and Power Engineering, Huazhong University of Science and Technology (HUST), Wuhan 430074, People’s Republic of China; 3 School of Energy and Power Engineering, Huazhong University of Science and Technology (HUST), Wuhan 430074, People’s Republic of China; 4 Department of Aerospace and Mechanical Engineering, University of Notre Dame, Notre Dame, Indiana 46556, USA

## Abstract

Both electron and phonon transport properties of single layer MoS_2_ (SLMoS_2_) are studied. Based on first-principles calculations, the electrical conductivity of SLMoS_2_ is calculated by Boltzmann equations. The thermal conductivity of SLMoS_2_ is calculated to be as high as 116.8 Wm^−1^K^−1^ by equilibrium molecular dynamics simulations. The predicted value of *ZT* is as high as 0.11 at 500 K. As the thermal conductivity could be reduced largely by phonon engineering, there should be a high possibility to enhance *ZT* in the SLMoS_2_-based materials.

Thermoelectric materials are essential for converting waste heat to electricity and solid-state cooling, which have attracted much attention recently^1^,^2^,^3^,^4^,^5^,^6^,^7^,^8^,^9^,^10^. The dimensionless figure of merit (*ZT*) is utilized to evaluate the efficiency of the thermoelectric conversion, defined as: 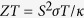, where *S* is the Seebeck coefficient, *σ* is the electrical conductivity, *T* is the absolute temperature, and *κ* is the total thermal conductivity. The *κ* is composed of electrons’ contribution (*κ*_*e*_) and phonons’ contribution (*κ*_*p*_). The *ZT* value for most commercial materials are around one, which is far below the critical value of three that is comparable with the traditional energy conversion in efficiency^3^. In the past two decades, nano-materials and nano-structured materials are expected to have excellent energy conversion efficiency due to the higher power factor (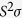)^11^,^12^ and lower *κ*_*p*_^13^,^14^,^15^, which are also known as the electron-crystal and phonon-glass.

The graphene, as the first two dimensional material, has extraordinary electronic property as well as super high thermal conductivity^16^. However, the pristine graphene, a semi-metal, has zero band gap and very small *S*^17^. Different from graphene, single layer MoS_2_ (SLMoS_2_) is a semiconductor and has a direct band-gap^18^, which enables its wide applications in electronic and optical devices, such as field effect transistor^19^.

Recently, some works have studied the electronic and phononic properties of SLMoS_2_. Eugene *et al.* have calculated the electronic structure of SLMoS_2_ which is compared with that of bulk MoS_2_^20^, and revealed the transition mechanism from the direct band gap of SLMoS_2_ to the indirect band gap of bulk MoS_2_. Emilio *et al.* have shown that, after applying compressive or tensile bi-axial strain, the electronic structure of SLMoS_2_ transitions from semiconductor to metal^21^. Li *et al.* calculated the intrinsic electrical transport and electron-phonon interaction properties of SLMoS_2_^22^. Moreover, the thermoelectric potential of SLMoS_2_ has been explored and a maximum *ZT*, at room temperature, is obtained as 0.5 by Huang *et al.*^23^ using the ballistic model. The scatterings of electrons are not considered in their ballistic model, which should have led to an over-estimation of *ZT*. Fu *et al.* studied SLMoS_2_ ribbons and calculated the *ZT* value to be up to 3.4^24^. Besides theoretical predictions, Wu *et al.* has experimentally reported a value of *S* as 30 mV/K for SLMoS_2_^25^, which indicates an appealing potential for thermoelectric applications.

Besides electron properties, some works focused on the phonon properties of SLMoS_2_. The SLMoS_2_ nanoribbon has a low thermal conductivity due to the size effect. Jiang *et al.* claimed that *κ*_*p*_ of SLMoS_2_ nanoribbon was around 5 Wm^−1^K^−1^ at room temperature by molecular dynamics (MD) simulations^26^. Zhang *et al.* reported three results for SLMoS_2_ nanoribbons which were 1.35 Wm^−1^K^−1^ by equilibrium molecular dynamics (EMD)^27^, 23.2 Wm^−1^K^−1^ by non-equilibrium Green’s function^28^, and 26.2 Wm^−1^K^−1^ by Boltzmann transport equation^29^. However, there are also reports on the thermal conductivities for MoS_2_ with higher values. Li *et al.* predicts the *κ* as 83 Wm^−1^K^−1^ from *ab initio* calculations^30^. With high-quality sample, the *κ* of suspended few layers MoS_2_ has been measured as 52 Wm^−1^K^−1^ 31 and 35 Wm^−1^K^−1^ 32. Liu *et al.* claimed that the basal-plane thermal conductivity of single crystal MoS_2_ would be 85–110 Wm^−1^K^−1^ 33. There is not an agreement on the *κ* of SLMoS_2_, and it needs more works on this issue.

In this paper, both electron and phonon transport properties of SLMoS_2_ are studied (the structure as shown in Fig. 1). Based on the electronic band structure from first-principles calculations, the electrical conductivity of SLMoS_2_ is calculated by Boltzmann equations. Both the electronic structure and phonon dispersion relation are calculated. Together with *κ*_*p*_ calculated from classical EMD simulations, the thermoelectric properties are obtained. The results show that SLMoS_2_ is a promising material for thermoelectric engineering.

## Results and Discussions

The electronic band structure of SLMoS_2_ along the high-symmetry points in Brillouin zone is shown in Fig. 2(a). At the K point, there is a direct band gap as 1.86 eV which agrees well with previous calculations (1.69 ~ 1.98 eV)^20^,^21^,^22^,^23^,^34^,^35^. Another characteristic in the SLMoS_2_ band structure is that there is a Q valley along the Γ-K path. The Q valley yields a larger effective mass than the K valley, which leads to strong electron-phonon interactions in MoS_2_ at this point^22^. The large effective mass of carriers and multi-valleys band structure are favorable for a high *ZT*^36^. As shown in the density of state (DOS) electrons (Fig. 2), there are sharp gradients at the edges of both conduction and valence band and several peaks near band edges, due to the quantum size effects in the 2D structure, which may enhance *ZT* as the prediction of Mahan and Sofo^12^.

We made a full calculation of the thermoelectric properties of SLMoS_2_ at 300 K, 400 K and 500 K. As shown in Fig. 3(a,b), κ_e_ and σ increase as the increasing of carrier concentration (*n*_*e*_). When the Fermi level is in the band gap, *n*_*e*_ and *σ* is much smaller. As the Fermi level moves up into the conduction band, *n*_*e*_ and *σ* increases quickly (more details shown in Fig. S4 and S6 in supporting information). Shown in Fig. 3(a), the Seebeck coefficient has a large value and decreases with the increase of *n*_*e*_. The Fermi level for *ZT* peak locates around the first DOS peak, and this is consistent with the prediction that a delta DOS would result in an optimum *ZT*^12^. It leads to a power factor as high as several hundreds of μWcm^−1^K^−2^ (shown in Fig. 3(c)), which is compared with those of high *ZT* thermoelectric materials, such as BiTe^37^ and PbTe^38^.

The phonon dispersion relation of SLMoS_2_ is also calculated and shown in Fig. 2(b). In the vicinity of Γ point, the out-of-plane transverse acoustic branch (ZA) has a quadratic relation, both the transversal acoustic branch (TA) and longitudinal acoustic branch (LA) have linear relations. The group velocities at Γ point along Γ-M direction are around 667.5 m/s (TA) and 1080.2 m/s (LA), which are much smaller than the group velocities in graphene^39^, as 3743 m/s (TA) and 5953 m/s (LA).

For semiconductors, the thermal conductivity is mainly contributed by phonons (*κ*_*p*_). We calculated *κ*_*p*_ by EMD and show in Fig. 4. The *κ*_*p*_ of SLMoS_2_ exhibits a size dependence on the simulation cell and reaches a converged value when the simulation cell is larger than 8 × 8 × 1 units^3^ (8.66 × 7.50 × 0.616 nm^3^) (Fig. 4(a)). A weak anisotropy is observed in thermal conductivities along armchair and zigzag direction. The average value of *κ*_*p*_ along armchair and zigzag directions is 116.8 Wm^−1^K^−1^ for simulation cell as large as 32 × 32 × 1 units^3^ (34.7 × 30.0 × 0.616 nm^3^) at 300K. In Fig. 4(b), the *κ*_*p*_ of SLMoS_2_ decreases with the increasing temperature (79.6 Wm^−1^K^−1^ and 52.9 Wm^−1^K^−1^ at 400 K and 500 K, respectively), because there are more three phonon Umklapp scatterings for high temperature. A lower *κ*_*p*_ is good for enhancing thermoelectric properties.

Comparing with previous results (details in Table 1), we obtained a maximum value of *κ*_*p*_ of SLMoS_2_. Some of these works focused on the SLMoS_2_ nanoribbons^26^,^27^,^29^ which have very low thermal conductivities, because the phonon confinement effect in nanostructures^40^,^41^. Using the same empirical potential in MD simulations, our results for SLMoS_2_ is around 20 times larger than that of nanoribbon with 34.6 × 30 × 0.61 nm^3^ in size. Besides, due to the interlayer coupling by van Der Waals forces^42^, the multilayer structures^31^,^33^ should be lower than the single layer^27^,^28^,^30^,^32^ in thermal conductivity. Due to the absence of impurities, defects and interlayer scatterings in MD simulations, the *κ*_*p*_ of SLMoS_2_ is a little higher than the measurements of bulk multilayer SLMoS_2_^33^, 85 ~ 110 Wm^−1^K^−1^. Our value is comparable to the result predicted from *ab initio* calculation^30^ , where stated that the lower bound of *κ*_*p*_ as 83 Wm^−1^K^−1^ at 300 K in the considering of phonon scatters and the simplification in calculation of BTE model. Another advantage for our results is that both the nonequilibrium Green’s function calculation^28^ and the Boltzmann transport equation^29^ adopt artificial relaxation time approximations for phonon-phonon Umklapp scatterings, which is not required in the MD simulations.

Generally, there are two types of commonly used MD simulation methods, EMD and non-equilibrium MD (NEMD). The EMD is better than NEMD in predicting a bulk structure by applying the periodic boundary condition, because NEMD need impose artificial heat bath and use the extrapolating method. However, NEMD gets the advantage of EMD in predicting a structure with a finite size. There is a EMD report which showed a low value of κ_p_ of SLMoS_2_ as 1.35 Wm^−1^K^−1^^27^ The potential functions used in Ref. [27] are determined by tight-binding quantum chemistry calculations and used to reproduce the crystal structure and Raman spectrum. The empirical potential function is important to obtain a reliable value of thermal conductivity. Differently, the Stillinger-Weber potential^26^ ,used in this work, can reproduce a better phonon dispersion relations, which will describe the heat transfer properties with a better reliability (details in supporting information).

With the above calculations of electron and phonon properties, *ZT* profiles can be obtained and are shown in Fig. 3(d). There is a parabolic tendency for *ZT* in the whole carrier concentration range. The optimized *ZT* values are 0.04, 0.07, and 0.11 for 300 K, 400 K and 500 K, respectively. These values get bigger as temperature increases because of the improved power factors and the reduced thermal conductivity. These optimized *ZT* values correspond to the situations where the Fermi level moves up to the first peak in the conduction band.

As shown in Table 1, we list some recently results on thermoelectric properties of different SLMoS_2_ structures. The value of *ZT* is in the same order of Ref. [23] and one order smaller than that of SLMoS_2_ ribbon. Our results of *κ*_*p*_ is higher than others. As shown in Fig. 4(a), our results of *κ*_*p*_ overcome the size confinement effect and corresponds to an infinite SLMoS_2_ sheet. Moreover, different from NEMD, it does not need the assumption of linear relationship between 1/*κ*_*p*_ and 1/L.

Compared to nanoporous silicon analyzed by Lee^43^, we get the similar *ZT* trend and magnitude of these transport values. As shown in Fig. 3(c), the power factor of SLMoS_2_ is larger than that of nanoporous silicon. The large power factor of SLMoS_2_ comes from a larger intrinsic *σ* and a comparable *S*. It indicates that the SLMoS_2_ has comparable electron properties as the optimized nanoporous silicon. However, due to the high *κ*_*e*_ and *κ*_*p*_, the SLMoS_2_ has a modest *ZT* value. It is also worth noting that the *ZT* value here is smaller than the prediction from ballistic models by Huang *et al.* where neglects the phonon scatterings^23^.

Although the predicted *ZT* value of SLMoS_2_ is not over one, SLMoS_2_-based materials may be a good candidate for thermoelectric application. Our results show that SLMoS_2_ has a much higher thermal conductivity (~116 Wm^−1^K^−1^, at 300 K) than other thermoelectric materials (on the order of 1 Wm^−1^K^−1^)^6^,^44^. The higher thermal conductivity makes a bigger room for thermal conductivity reduction by phonon engineering. There are some conventional ways to reduce thermal conductivity by phonon engineering, such as isotope doping^45^, nanoporous structure^14^,^43^,^46^,^47^, nanoribbons^48^, or folding^49^ etc. The mechanism is to introduce more phonon scatterings which can shorten phonon mean free paths. For example, bulk silicon has a *ZT* value as low as 0.003. Then, with phonon engineering, Si-based nanomaterials, such as Si nanowires^45^,^50^, nanoporous Si^14^,^43^,^46^,^47^, and nanostructured Si^51^, may reach a two orders larger *ZT*. Another inspiration example is the graphene. The high pristine thermal conductivity of graphene can be reduced largely by phonon engineering^48^,^49^,^52^ which make a *ZT* as high as 3^52^

The values of *ZT* and power factor of SLMoS_2_ are much higher than those of silicon and graphene. With a reduced thermal conductivity and kept electron transport properties, the values of *ZT* of SLMoS2-based materials may be larger than one. Generally, a side-effect of phonon engineering is the reduction of power factor. However, the side-effect is not obvious because the mean free paths of electrons are around two orders smaller than that of phonons, such as what is shown in the recent thermoelectric results on Si phononic crystals^47^

## Conclusion

The thermoelectric properties of SLMoS_2_ are explored using theoretical calculations. The electronic structure and phonon dispersion relation are calculated using DFT calculations. Combined with molecule dynamics simulations and Boltzmann equations, thermoelectric properties are predicted as a function of carrier concentration at room temperature. With the lattice thermal conductivity as 116.8 Wm^−1^K^−1^, 79.6 Wm^−1^K^−1^, and 52.9 Wm^−1^K^−1^, the optimized *ZT* of SLMoS_2_ is found to be of 0.04, 0.07 and 0.11 at 300 K, 400 K and 500 K, respectively. As SLMoS_2_ has a higher *ZT* than other pristine structure, like silicon and graphene, there will be a big room to enhance *ZT* in SLMoS_2_-based materials by the developing phonon-engineering.

## Methods

To calculate electronic properties, the first-principles calculation is implemented by QUANTUM ESPRESSO in the frame of density functional theory (DFT)^53^. The local density approximation (LDA) is used in the exchange-correlation approximation while the semi-core valence for molybdenum is considered with the Goedecker-Hartwigsen-Hutter-Tetter method^54^. The wave-functions in electronic calculation are cut off at 160 Ry, and the irreducible Brillouin zone is sampled with a 16 × 16 × 1 Monkhorst-Pack grid.

The hexagon primitive cell is used to structure relaxation and property prediction in DFT calculation. Structure relaxation for SLMoS_2_ yields lattice constant of about 3.13 Å, consistent with previous predictions of 3.12–3.16 Å^20^,^21^,^22^. For the consistency of property evaluation, the thickness of SLMoS_2_ is assumed to be 6.16 Å – the same as that of the single-sheet in bulk MoS_2_^55^. The calculations on both electrons and phonons are based on this optimized structure.

In the calculations of transport coefficients, a k-point mesh as 28 × 28 × 1 (denser enough to obtain converged results) is used over the irreducible Brillouin zone. With the assumption of constant relaxation time, the transport coefficient for electrons can be calculated using BoltzTrap^56^ which solves Boltzmann transport equation (more details in supporting information).

**Figure 1:**
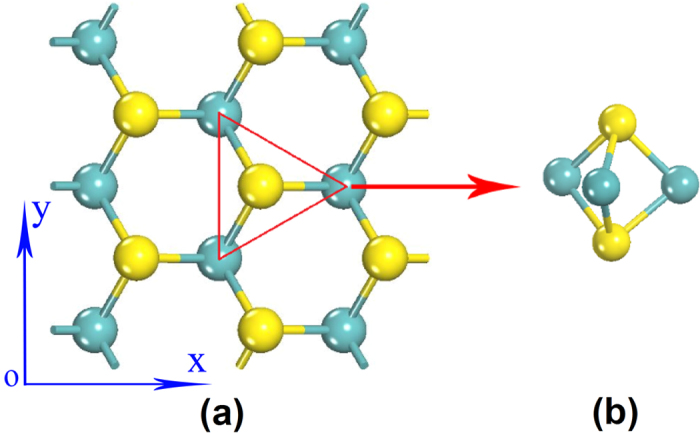
The structure of single layer MoS_2_. (**a**) The top view, a hexagonal lattice structure. (**b**) The side view of the inset triangle. Each sulfur atom has three molybdenum atoms as its first nearest neighbor atom. Each molybdenum atom has six sulfur atoms as its first nearest neighbor atom.

**Figure 2:**
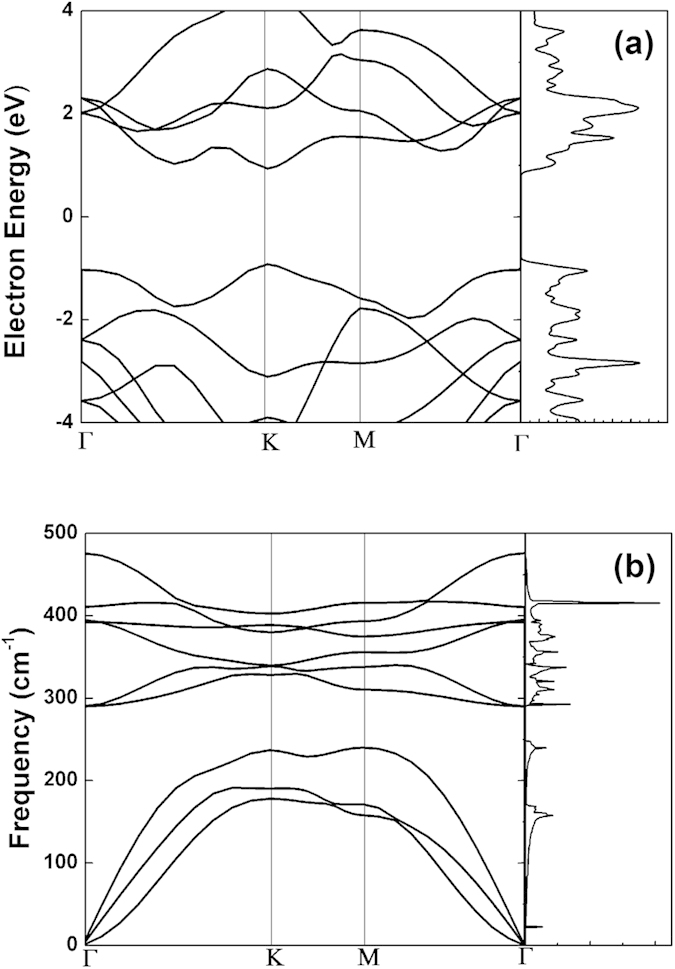
(**a**) Electron band structure and density of state along the symmetry line. The Fermi energy is set in the middle of the gap. (**b**) The phonon dispersion for SLMoS_2_ and the phonon density of state in the whole Brillouin zone.

**Figure 3:**
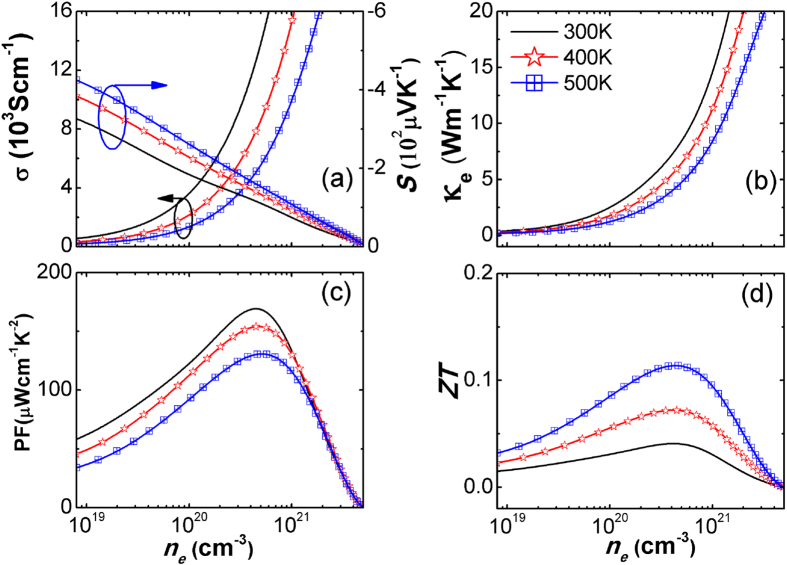
The thermoelectric transport properties of n-type SLMoS_2_ at 300K, 400K and 500K. (**a**) The electrical conductivity and Seebeck coefficient; (**b**) The electrical thermal conductivity; (**c**) The power factor; (**d**) The figure of merit. The thermoelectric transport properties of p-type is shown in supporting information.

**Figure 4:**
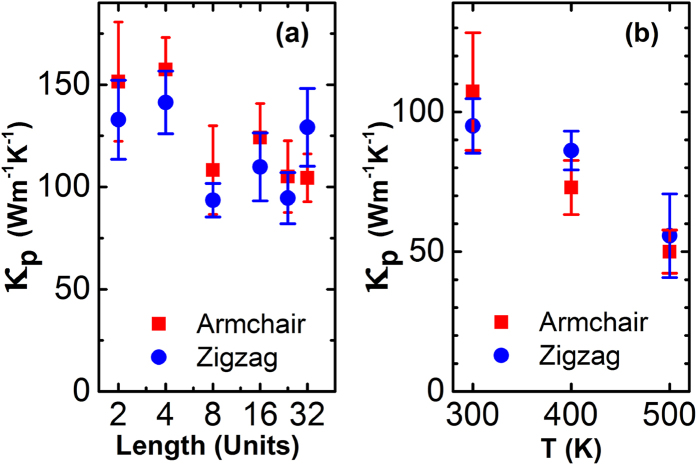
(**a**) The dependence of thermal conductivity (κ_p_) of SLMoS_2_ upon the size of simulation cell. The size of simulation cell size equals the length (L) times a supercell (1.083 × 0.938 × 0.616 nm^3^). (**b**) The thermal conductivity of SLMoS_2_ for three different temperature as 300K, 400K and 500K, respectively.

The thermal conductivity of SLMoS_2_, *κ*_*p*_, is calculated by EMD with the Green-Kubo approach^57^. All the simulations are carried out utilizing the LAMMPS software package^58^. The Stillinger-Weber potential with parameters fitted by Jiang *et al.*^26^ is adopted in our simulations. The SLMoS_2_ film is constructed by periodic arrangement of supercell illustrated in Fig. 1, and the sizes of 1 × 1 × 1 units^3^ supercell corresponds to 1.083 × 0.938 × 0.616 nm^3^. To study the finite size effect on thermal conductivities, we calculated the simulation cells with the volumes from 2 × 2 × 1 to 32 × 32 × 1 units^3^ at room temperature (more details in supporting information).

## Additional Information

**How to cite this article**: Jin, Z. *et al.* A Revisit to High Thermoelectric Performance of Single-layer MoS_2_. *Sci. Rep.* **5**, 18342; doi: 10.1038/srep18342 (2015).

## Supplementary Material

Supplementary Information

## Figures and Tables

**Table 1:** The comparison of thermoelectric properties for different MoS_2_ structures, including single layer (SL), few layers (FL), single layer ribbon (SLR), and bulk MoS_2_.

Struct.& Ref.	Method	*T*(K)	Carrier type	*σ*(Scm^−1^)	*S*(μVK^−1^)	*κ*_*e*_	*κ*_*ph*_	*ZT*
						(Wm^−1^K^−1^)		
SL	DFT + BTE + MD	300	n	14625	−110	8.94	116.8	0.04
			p	16957	72.9	11.39		0.02
		500	n	11714	−161	9.69	52.9	0.26
			p	8853	150	8.40		0.16
SL^23^	DFT + Ballistic model	300	n	54	−202	0.021	0.243	0.25
			p	108	215	0.040	0.244	0.53
SLR^24^,^27^	DFT + BTE + MD	300	n	7770	−204	2.89	1.02	2.5
			p	14300	223	5.20		3.4
SL CVD^25^	Experiment	300	–	–	≤30000	–	–	–
SL FET^59^	Experiment	300	–	–	400–100000	–	–	–
Bulk^60^	Experiment	90–873	–	–	500–700	–	–	–
SL^27^	EMD	300	–	–	–	–	1.35	–
SL^30^	DFT + BTE		–	–	–	–	>83	–
SL^28^	DFT + NEGF		–	–	–	–	23.2	–
SLR^29^	DFT + BTE		–	–	–	–	26.2	–
SLR^26^	NEMD		–	–	–	–	5	–
FL^31^	Experiment		–	–	–	–	52	–
SL^32^	Experiment		–	–	–	–	35.4	–
Bulk^33^	Experiment		–	–	–	–	85– 110	–
