# Supplementary material for: A Revisit to High Thermoelectric Performance of Single-layer MoS2
Source: Sci Rep. 2015 Dec 17;5:18342. doi: 10.1038/srep18342 (PMC4683674; doi:10.1038/srep18342)
Supplement: Supplementary Information [file srep18342-s1.pdf]

Supporting Information

A Revisit to High Thermoelectric Performance of Single-layer MoS_2

Zelin Jin[1,2, #], Quanwen Liao[3, #], Haisheng Fang[1,2,*], Zhichun Liu[3,*], Wei Liu[3], Zhidong Ding[2], Tengfei Luo[4], Nuo Yang[1,2,*]

[1] State Key Laboratory of Coal Combustion, Huazhong University of Science and Technology (HUST), Wuhan 430074, People's Republic of China

[2] Nano Interface Center for Energy (NICE), School of Energy and Power Engineering, Huazhong University of Science and Technology (HUST), Wuhan 430074, People's Republic of China

[3] School of Energy and Power Engineering, Huazhong University of Science and Technology (HUST), Wuhan 430074, People's Republic of China

[4] Department of Aerospace and Mechanical Engineering, University of Notre Dame,

Notre Dame, Indiana 46556, USA.

[#] Z. J. and Q. L. contributed equally to this work.

* Corresponding authors: N.Y. (nuo@hust.edu.cn), H. F. (hafang@hust.edu.cn), and Z.L. (zcliu@hust.edu.cn)

I. Molecular dynamics (MD) simulation details

The lattice thermal conductivity of single-layer MoS_2 ($SLMoS_2$) is calculated by equilibrium molecular dynamics [S1]. Thermal conductivity is derived from the Green-Kubo formula [S2] as

$$\kappa = \frac{1}{3k_B T^2 V}\int_0^\infty < \vec{J}(\tau)\cdot\vec{J}(0) > d\tau \qquad \text{(S1)}$$

$$\vec{J}(\tau) = \sum_i \vec{v}_i \varepsilon_i + \frac{1}{2}\sum_{\text{ij } i\neq j}\vec{r}_{ij}(\vec{F}_{ij}\cdot\vec{v}_i) + \sum_{ijk}\vec{r}_{ij}(\vec{F}_j(ijk)\cdot\vec{v}_j)$$

where κ is the thermal conductivity, k_B the Boltzmann constant, V the system volume, T the temperature, the angular bracket denoting ensemble average. $\vec{v}_i$, ε_i , and $\vec{r}_i$ are the velocity vector, the site energy, and the position vector of atom respectively. The distance between atom i and atom j is $\vec{r}_{ij} = \vec{r}_j - \vec{r}_i$. $\vec{F}_{ij}$ and $\vec{F}_j(ijk)$ denote the two-body and the three-body force, respectively.

The MD simulations are carried out utilizing LAMMPS software package [S3]. The Stillinger-Weber potential, whose parameters are fitted using GULP by Jiang *et al.* [S4], is adopted in our simulations (shown in Table S1). An appropriate empirical potential is fundamental to obtain a reliable MD calculation result. The phonon dispersion relations, which are extracted from two different empirical potentials for $SLMoS_2$, are compared with the results from DFT calculation, as shown in Fig. S1. The potential function used in this work provides a better reproduction of the phonon dispersion, which will assure a more reliable result on thermal conductivity.

Different from previous works on MoS_2 ribbons [S5], our MoS_2 model is a $SLMoS_2$ sheet. The periodic boundary conditions are applied along the two in-plane directions. There is no inter-

layer interaction due to the single layer structure. Therefore, in the simulations, the inter-layer van der Waals interactions are not taken into account. The two in-plane directions studied correspond to the armchair direction and the zigzag direction.

Generally, the temperature in MD simulation, T_{MD}, is calculated from the kinetic energy of atoms according to the Boltzmann distribution:

$$\langle E \rangle = \sum_{i=1}^{N} \frac{1}{2} m v_i^2 = \frac{3}{2} N k_B T_{MD} \tag{S2}$$

where $\langle E \rangle$ is the total kinetic energy, m the atomic mass, and N the number of particles in the system, respectively.

The velocity Verlet algorithm is employed to integrate equations of motion, and the time step is 0.5 fs. Initially, the Nose-Hoover heat reservoir is used to equilibrate the system at 300 K for 5×10^5 time steps (250ps). Then, simulations run in the *NPT* ensemble for 500 ps (10^6 time steps) to relax the structure. After relaxation, the converged values of both the cell size and the potential energy are obtained, which makes sure that there is no stress or strain effects. Then, the structure runs another 5 ns under NVE ensemble for relaxation. Now the system is ready for heat flux recording. The heat current vector is calculated and recorded each 2.5 fs for 8×10^6 time steps to obtain the autocorrelation function and thermal conductivity.

In Fig. S2, the black curve shows the normalized heat current autocorrelation function (HCACF) used in Green-Kubo formula to calculate thermal conductivity, where the side length of simulation cell is 8 units and the temperature is 300 K. Additionally, the blue curve shows the thermal conductivity which is an integration of the HFACF. The thermal conductivity converges

to 93.55 $Wm^{-1}K^{-1}$ after around 220 ps due to the decay of the HFACF. The final thermal conductivity is the mean value of twelve realizations with different initial conditions.

II. Finite size effect in simulations

When using Green Kubo formula to calculate thermal conductivity, the finite size effect could arise if the simulation cell is not sufficiently large [S2]. Fig. 4 shows the thermal conductivity of $SLMoS_2$ at room temperature with different cell sizes, from $2 \times 2 \times 1$ to $32 \times 32 \times 1$ $unit^3$. The thermal conductivity shows a strong dependence on the size when it's smaller than $8 \times 8 \times 1$ $unit^3$. However, it changes little when the size is larger. The simulation cell in our calculations is selected as large as $32 \times 32 \times 1$ $units^3$ ($34.7 \times 30.0 \times 0.616$ nm^3) which is large enough to overcome the finite size effect.

III. The anharmonic effects of the empirical potential function

In MD simulations, the inter-atomic potential parameters are fundamental to the accuracy of the thermal conductivity calculations. The inter-atomic potential parameters herein predict the thermal expansion coefficient to be 4.85×10^{-6} K^{-1} at room temperature, which is lower than the results of Huang *et al* [S6] and C. Sevik [S7] that is about 6.74×10^{-6} K^{-1} from the predictions of the first principles. It means that this empirical potential function somewhere underestimates the real anharmonicity and phonon-phonon scatterings. That is, the thermal conductivity would be overestimated in our work. Moreover, an overvalued thermal conductivity results in an underestimated *ZT*.

IV. Electron transport properties calculation details

We compared our DFT calculation in electron and phonon dispersion curves with previous results. Fig. S3 shows the electron and phonon band structures. The black solid lines are from our calculation, while the open square dots are the reference data coming from Ref. 22 of the main article. Our results agree well with the result in Ref. 22 of the main article.

The Boltzmann transport equation (BTE) is utilized to predict electronic transport properties. The assumptions, the constant relaxation time and the rigid band approximation[S8], are used in BTE calculation[S9]. These strategies in transport coefficients calculation have been verified through previous works[S8,S10]. With constant relaxation time assumption, transport coefficients for electrons can be obtained by

$$\sigma = \mathrm{L}^{(0)} \tag{S3}$$

$$S = -(1/eT)\sigma^{-1}\mathrm{L}^{(1)} \tag{S4}$$

$$\kappa_e = 1/(e^2T)\,(\mathrm{L}^{(2)} - \mathrm{L}^{(1)}\sigma\mathrm{L}^{(1)}) \tag{S5}$$

where $\mathrm{L}^{(\alpha)}$ is defined as :

$$\mathrm{L}^{(\alpha)} = e^2\tau\sum_n \int \frac{d\vec{k}}{4\pi^3}\left(-\frac{\partial f(\varepsilon_{n\vec{k}})}{\partial\varepsilon_{n\vec{k}}}\right)\overrightarrow{v_{n\vec{k}}}\,\overrightarrow{v_{n\vec{k}}}(\varepsilon_{n\vec{k}} - \mu)^{\alpha} \tag{S6}$$

where, $\varepsilon_{n\vec{k}}$ is the energy eigenvalue of the nth band at points in the first Brillouin zone, $f(\varepsilon_{n\vec{k}})$ the Fermi-Dirac distribution function at temperature T, μ the chemical potential, τ the

relaxation time and $\overrightarrow{v_{n\vec{k}}}$ the group velocity, respectively. Our calculations are focused on the in-plane transport coefficients of the two-dimensional structure. The Boltzmann equation for transport coefficients is solved by BoltzTrap [S9].

The relaxation time is a key parameter in solving Boltzmann equation. For electronic transport in semiconductor, the relaxation time is mostly affected by scatterings, such as impurity, boundary, and phonons. The relationship between the relaxation time and mobility (μ) is defined as $\tau = m^* \mu / e$, where m^* is the effective mass. We get the average values of effective mass, $0.50m_0$ for electrons and $0.64m_0$ for holes, where m_0 is the mass of electron. That is, we can obtain the relaxation time based on the mobility.

Kim *et. al* predicted the mobility in MoS_2 both theoretically and experimentally [S11]. It shows that the experimental results are consistent with the theoretical predictions. In their theoretical model, the impurity scatterings, acoustic phonon scatterings, and optical scatterings are all taken into consideration, which produces comprehensive results. Therefore, their values of mobility are used in our calculation. However, they provide values of mobility below 300 K only. We obtain the mobility for higher temperature according to the reciprocal relationship[S12,S13]. The values of mobility for 300 K, 400 K and 500 K are fitted as 180.27 $cm^2V^{-1}s^{-1}$, 117.55 $cm^2V^{-1}s^{-1}$ and 79.92 $cm^2V^{-1}s^{-1}$, respectively. Besides, based on the results from Kaasbjerg *et. al* [S12], the mobility is not sensitive to carrier concentration. Therefore, it is assumed that the mobility and relaxation time are independent on the concentration in our calculation.

The relaxation time of n-type $SLMoS_2$ for 300 K, 400 K, and 500 K are fitted as 5.17×10^{-14} s, 3.37×10^{-14} s and 2.29×10^{-14} s, respectively. The values are in the range of the prediction based on deformation potential theory by Fan *et al.*[S14].

V. Thermoelectric property

With the calculated band structures, we can obtain the transport tensor of $SLMoS_2$. In Fig. S5, there is little difference for Seebeck coefficients along two in-plane orthogonal directions, x and y. So, average values are taken and the $SLMoS_2$ is treated as isotropic here.

As the Fermi level shifts from band gap to valence band, we can get the transport properties under p-type carrier concentration with rigid band approximation. Fig. S6 shows the thermoelectric properties when $SLMoS_2$ is p-type doped. Compared with the results of *ZT* for n-type $SLMoS_2$ (shown in Fig. 3), the p-type has smaller *ZT* values, because the n-type has bigger Seebeck coefficients.

REFERENCES

S1. Hoover, W. G. *et al.* Lennard-Jones triple-point bulk and shear viscosities. Green-Kubo theory, Hamiltonian mechanics, and nonequilibrium molecular dynamics. *Phys. Rev. A* **22**, 1690-1697, doi:10.1103/PhysRevA.22.1690 (1980).

S2. Yang, L. N., Yang, N. & Li, B. W. Reduction of Thermal Conductivity by Nanoscale 3D Phononic Crystal. *Sci Rep* **3**, 1143, doi:10.1038/Srep01143 (2013).

S3. Plimpton, S. Fast Parallel Algorithms for Short-Range Molecular Dynamics. *J. Comput. Phys.* **117**, 1-19, doi:10.1006/jcph.1995.1039 (1995).

S4. Jiang, J. W., Park, H. S. & Rabczuk, T. Molecular dynamics simulations of single-layer molybdenum disulphide (MoS2): Stillinger-Weber parametrization, mechanical properties, and thermal conductivity. *J. Appl. Phys.* **114**, 064307, doi:10.1063/1.4818414 (2013).

S5. Jiang, J. W., Zhuang, X. Y. & Rabczuk, T. Orientation Dependent Thermal Conductance in Single-Layer MoS2. *Sci Rep* **3**, 4, doi:10.1038/srep02209 (2013).

S6. Huang, L. F., Gong, P. L. & Zeng, Z. Correlation between structure, phonon spectra, thermal expansion, and thermomechanics of single-layer MoS2. *Phys. Rev. B* **90**, 045409, doi:10.1103/PhysRevB.90.045409 (2014).

S7. Sevik, C. Assessment on lattice thermal properties of two-dimensional honeycomb structures: Graphene, h-BN, h-MoS2, and h-MoSe2. *Phys. Rev. B* **89**, 035422, doi:10.1103/PhysRevB.89.035422 (2014).

.

S8. Lee, M. S. & Mahanti, S. D. Validity of the rigid band approximation in the study of the thermopower of narrow band gap semiconductors. *Phys. Rev. B* **85**, doi:10.1103/PhysRevB.85.165149 (2012).

S9. Madsen, G. K. H. & Singh, D. J. BoltzTraP. A code for calculating band-structure dependent quantities. *Comput Phys Commu* **175**, 67-71, doi:10.1016/j.cpc.2006.03.007 (2006).

S10. Huang, B.-L. & Kaviany, M. Ab initio and molecular dynamics predictions for electron and phonon transport in bismuth telluride. *Phys. Rev. B* **77**, 125209, doi:10.1103/PhysRevB.77.125209 (2008).

S11. Kim, S. *et al.* High-mobility and low-power thin-film transistors based on multilayer MoS2 crystals. *Nat. commun.* **3**, 1101, doi:10.1038/ncomms2018 (2012).
S12. Kaasbjerg, K., Thygesen, K. S. & Jacobsen, K. W. Phonon-limited mobility in n-type single-layer MoS2 from first principles. *Phys. Rev. B* **85**, 115317, doi:10.1103/PhysRevB.85.115317 (2012).
S13. Kawamura, T. & Das Sarma, S. Phonon-scattering-limited electron mobilities inAlxGa1−xAs/GaAs heterojunctions. *Phys. Rev. B* **45**, 3612-3627, doi:10.1103/PhysRevB.45.3612 (1992).
S14. Fan, D. D. *et al.* MoS2 nanoribbons as promising thermoelectric materials. *Appl. Phys. Lett.* **105**, 133113, doi:10.1063/1.4897349 (2014).

Table S1. Stillinger-Weber potential parameters used in MD simulations. The two-body potential expression is $V_2 = \varepsilon A(B\sigma^p r_{ij}^{-p} - \sigma^q r_{ij}^{-q})e^{[\sigma(r_{ij}-a\sigma)^{-1}]}$. The three-body potential expression is $V_3 = \varepsilon\lambda e[\gamma\sigma(r_{ij} - a\sigma)^{-1} + \gamma\sigma(r_{jk} - a\sigma)^{-1}](\cos\theta_{jik} - \cos\theta_0)^2$. Energy parameters are in the unit of eV. The parameters of size are in the unit of Å.

	ε	σ	a	λ	γ	cos θ_0	A	B	p	q	tol
Mo-S-S	6.0672	0.7590	4.1634	1.0801	0.8568	0.1525	1.0	45.4357	4	0	0.0
S-Mo-Mo	6.0672	0.7590	4.1634	1.0801	0.8568	0.1525	1.0	45.4357	4	0	0.0
Mo-Mo-Mo	3.5040	0.6097	7.0034	0.0	0.0	0.0	1.0	181.8799	4	0	0.0
S-S-S	0.4651	0.6501	5.7837	0.0	0.0	0.0	1.0	125.0923	4	0	0.0

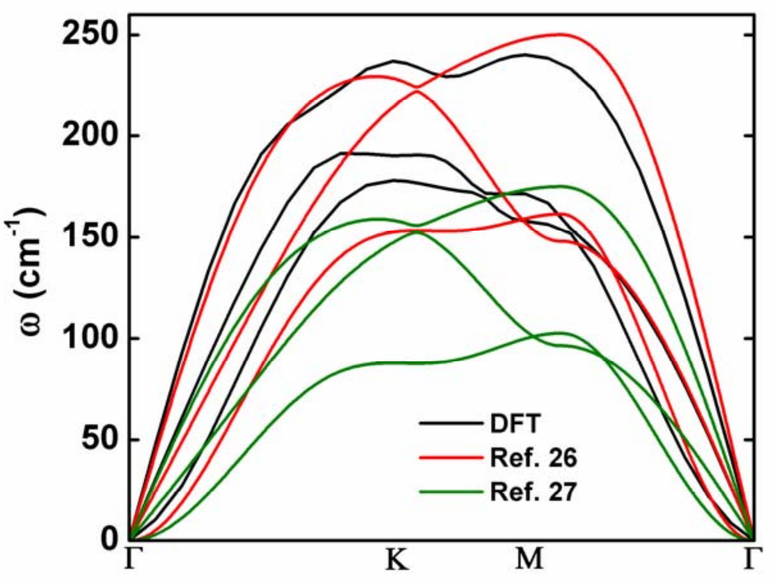

Fig. S1 The comparison of the acoustic phonon dispersion curves from DFT and the empirical potential functions implemented in Ref. 26 and Ref. 27 of the main article.

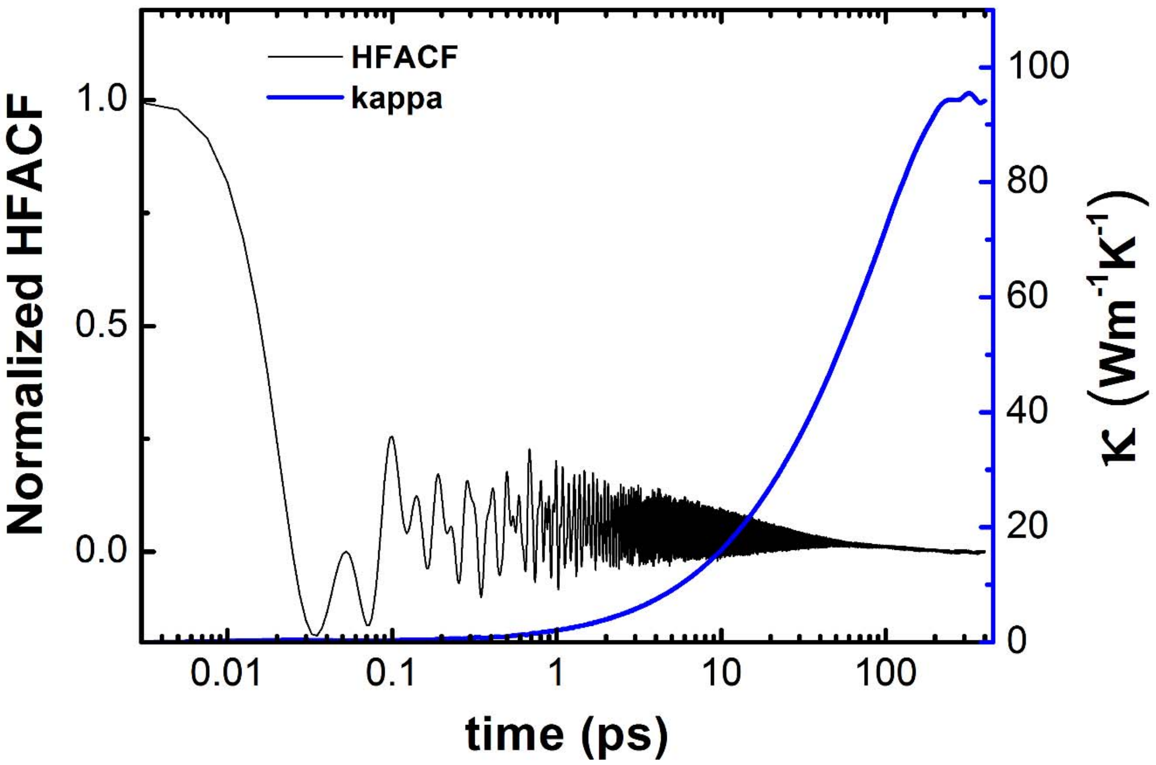

Fig. S2 The Normalized heat flux autocorrelation function (HFACF) and thermal conductivity (κ) along the y direction of $SLMoS_2$ versus correlation time. The side length of simulation cell is 8 units. The figure shows, after 220 ps, the HFACF decays close to zero and the thermal conductivity converges.

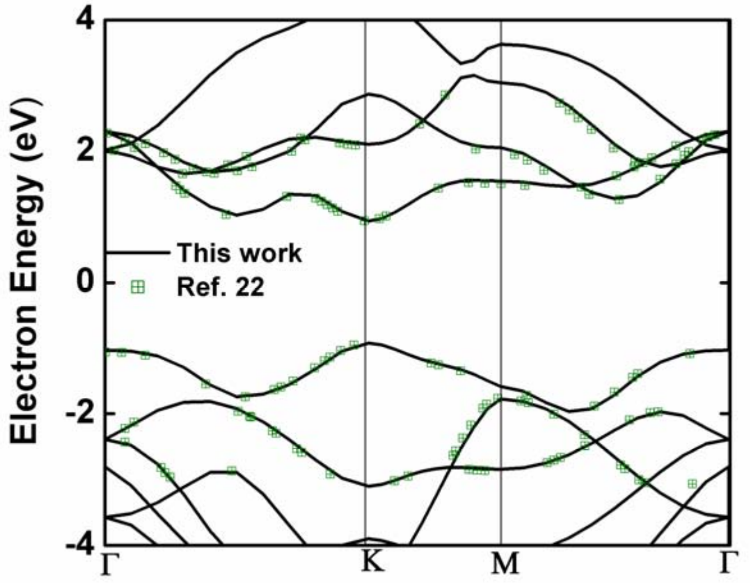

(a)

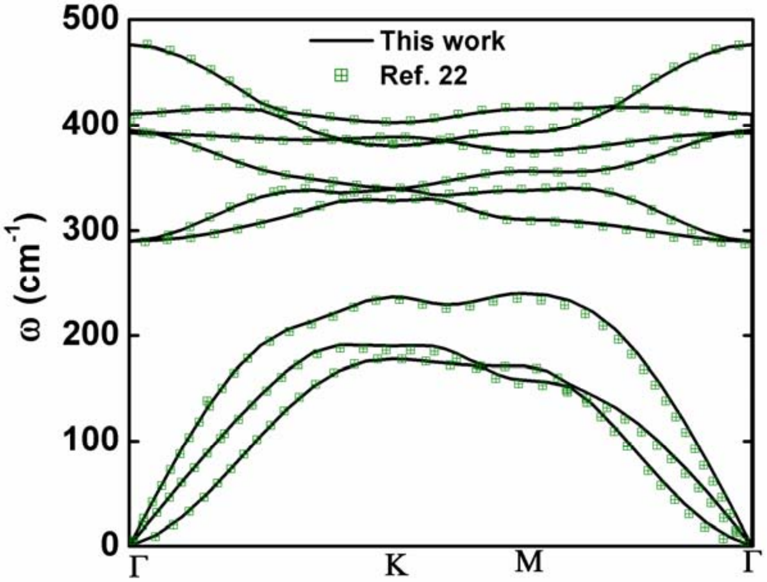

(b)

Fig. S3 (a) The electron band structures come from our calculation and those in Ref. 22 of the main article. (b) The phonon dispersion curves come from our calculation and those in Ref. 22 of the main article.

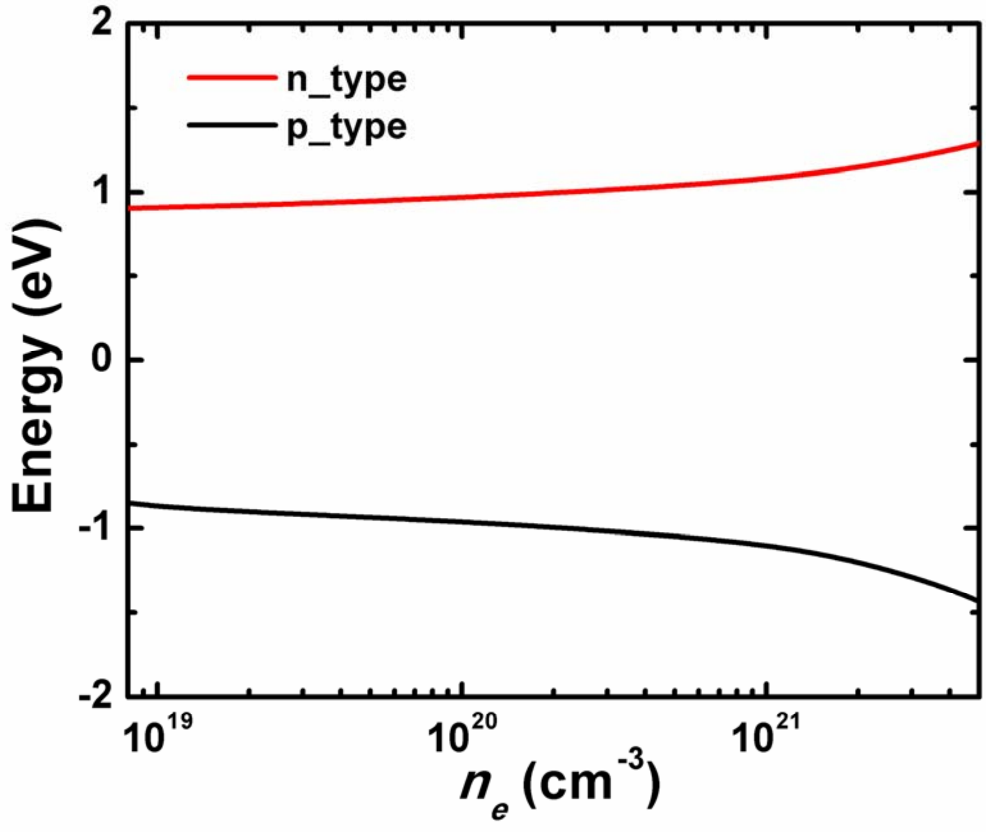

Fig. S4 The chemical potential versus the carrier concentration.

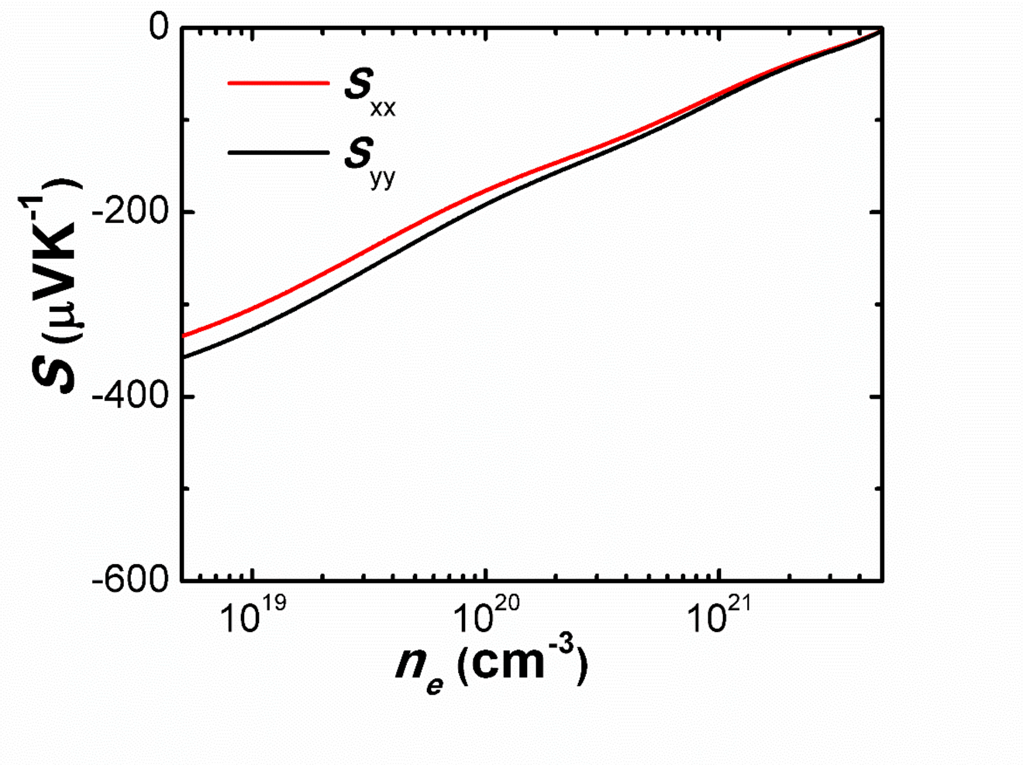

Fig. S5 The Seebeck coefficients of SLMoS_2 along two in-plane orthogonal directions, x and y. .

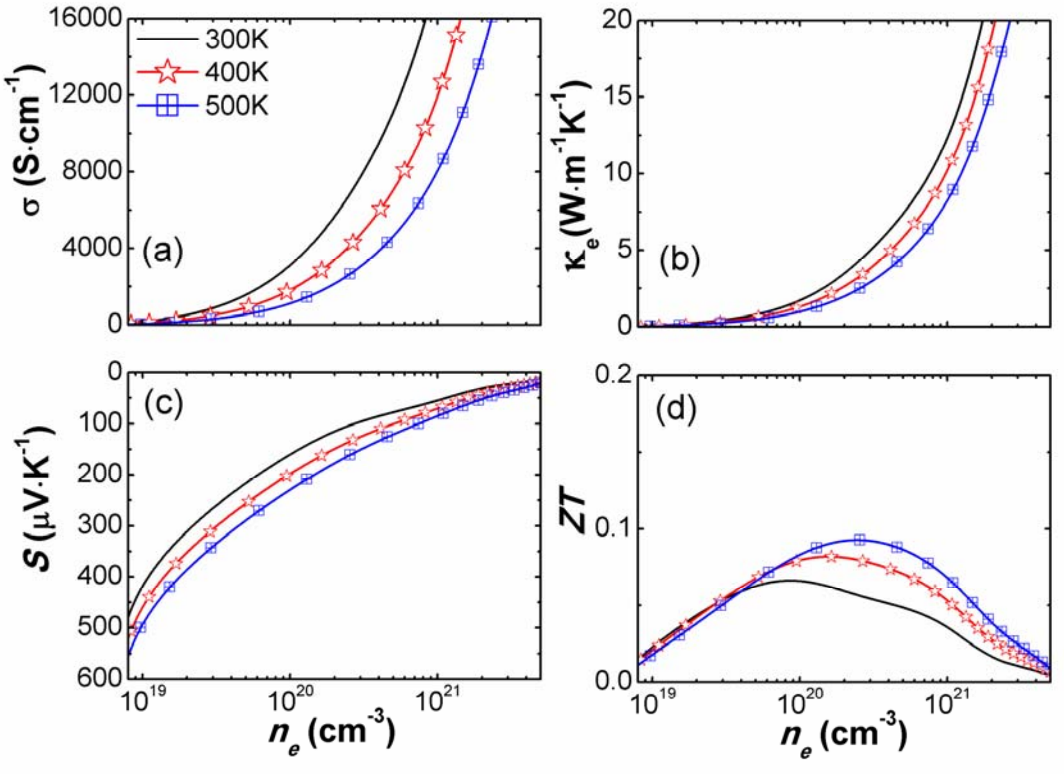

Fig. S6 The thermoelectric transport properties of p-type $SLMoS_2$ at 300 K, 400 K and 500 K. (a) The electrical conductivity; (b) The electronic thermal conductivity; (c) The Seebeck coefficient; (d) The figure of merit.
